# Fabrication of 3D Printed Polylactic Acid/Polycaprolactone Nanocomposites with Favorable Thermo-Responsive Cyclic Shape Memory Effects, and Crystallization and Mechanical Properties

**DOI:** 10.3390/polym15061533

**Published:** 2023-03-20

**Authors:** Hao Liu, Chengdi Li, Simin Chen, Ping Chen, Jinbo Li, Huihua Jian, Guoyi Guo, Xiao Chen, Xiaofeng Zhu, Jun Wu

**Affiliations:** Xinyu Key Laboratory of Materials Technology and Application for Intelligent Manufacturing, School of Mechanical and Electrical Engineering, Xinyu University, Xinyu 338004, China

**Keywords:** fused filament fabrication, interface compatibilizer, hybrid nanofillers, nanocomposites, thermo-responsive shape memory effects

## Abstract

In this work, 3D printed polylactic acid (PLA)/polycaprolactone (PCL) nanocomposites with favorable thermo-responsive cyclic shape memory effects (SMEs) and crystallization and mechanical properties were fabricated using a two-step method. First, an isocyanate-terminated PCL diol (PCL-NCO) was synthesized through the reaction between isocyanate groups of hexamethylene diisocyanate and active hydroxyl groups of PCL diol, and its physicochemical properties were characterized. A PLA/PCL blend with a PCL content of 50 wt% was fabricated via fused filament fabrication (FFF) 3D printing, and the influence of the PCL-NCO on the SME of the PLA/PCL blend was studied. The results indicated that the PCL-NCO significantly improved the cyclic shape memory performance of 3D printed PLA/PCL blends and was proved to be an effective interface compatibilizer for the blend system. Subsequently, the structure and properties of 3D printed PLA/PCL nanocomposites were investigated in detail by adding cellulose nanocrystal-organic montmorillonite (CNC-OMMT) hybrid nanofillers with different contents. It was found that the hybrid nanofillers greatly enhanced crystallization and mechanical properties of the nanocomposites due to adequate dispersion. The modification of the PLA/PCL blend and the preparation of the 3D printed nanocomposite can not only prolong the service life of a shape memory polymer product, but also broaden its application scope in advanced fields.

## 1. Introduction

Over the past few decades, there has been continued interest in shape memory polymers (SMPs), and efforts have been devoted to the development of novel SMPs and their composites [1,2,3]. As a stimulus-responsive polymer, SMP can recover from a temporary shape to its original shape under external stimuli (such as light, electricity, heat, magnetism, etc.). Generally, the SMP contains a stable polymer network and a reversible stimulus-sensitive switch. The polymer network determines the permanent shape, and the reversible switch, which can be a crystalline domain [4], amorphous domain [5], or hydrogen bonding network [6,7], is responsible for fixing and releasing the temporary shape. With the introduction of nanomaterials, SMP nanocomposites have large recoverable deformation, high mechanical properties, and an adequate cyclic deformation performance. Because of these unique characteristics, the SMPs and SMP nanocomposites have promising applications in aerospace engineering [8], biomedical devices [9], flexible electronics [10], and soft robotics [11].

Today, the emerging 3D printing technologies provide a new route for the fabrication of SMPs and SMP nanocomposites [2,12,13]. Three-dimensional printing breaks away from conventional manufacturing and changes our understanding of manufacturing. Due to design flexibility, a short development cycle, and low production cost, 3D printing has gained popularity in the field of complex parts manufacturing. Fused filament fabrication (FFF) 3D printing is one of the most widely used rapid prototyping technologies [14,15]. In our previous work, polylactic acid (PLA)/polycaprolactone (PCL) blends with various mass ratios were prepared via the FFF [16]. The influences of composition, interface, and morphology of the blends on their thermo-responsive shape memory effects (SMEs) were systematically studied, and the corresponding SME mechanism was elucidated. Additionally, based on the optimization of printing parameters, a tunable and favorable SME was achieved by precisely and effectively regulating the internal construction of the 3D printed product. Finally, thermo-responsive shape memory behavior of the 3D printed PLA/PCL SMP was verified under practical conditions. This work demonstrated the advantages of 3D printing in the fabrication of shape memory materials.

As far as the SMP composed of a PLA/PCL blend, fixed phase PLA and reversible phase PCL exhibit a phase-separated microstructure, and the thermal transition temperature regions of the two phases are far apart. These provide necessary prerequisites for thermally responsive SME [17]. However, the PLA/PCL blend system also suffers from some drawbacks, such as low modulus and strength, and poor thermo-mechanical cycling performance [18]. Appropriate chemical modification for the PLA/PCL blend [19] and further introduction of nanofillers to prepare SMP nanocomposites [20] are important approaches to overcome these defects.

With regard to the modification of the polyester blend, a low-cost, high-efficiency method is reactive blending/extrusion, i.e., the addition of a specific compatibilizer during thermo-mechanical processing [21,22]. The compatibilizer with active groups acts as “links” to improve the interfacial properties between polymer components. Davide and Annamaria et al. introduced ethyl ester L-lysine tri-isocyanate (LTI) into the PLA/PCL blend [23,24]. They found that the isocyanate group of the LTI reacted with the hydroxyl group at the end of the polyester chain to form a graft-crosslinked polyester-urethane-polyester structure in situ during the melt-mixing process. As a result, the LTI enhanced interfacial interaction between PLA and PCL, thereby significantly improving mechanical properties of the blend.

On the other hand, the addition of nanofillers could change the structure and properties of the PLA/PCL blend system. Cellulose nanocrystal (CNC) has been considered as an excellent reinforcing nanomaterial [25,26]. In a recently published report, novel CNC-organic montmorillonite (OMMT) hybrid nanoparticles with favorable thermal stability were constructed by electrostatic self-assembly [27]. Then, the hybrid nanoparticles were incorporated into a PLA matrix to prepare PLA nanocomposites by FFF 3D printing [28]. We found that the hybrid nanofillers had synergistic enhancement effects on mechanical, crystallization, and heat resistant properties of the nanocomposites due to dramatically improved dispersion of the hybrid nanofillers in the PLA matrix. Unfortunately, there were few reports on the influences of CNC-based hybrid nanofillers on the shape memory performance, crystallization, and mechanical properties of PLA/PCL nanocomposites.

In this work, isocyanate groups were introduced into a PCL diol using hexamethylene diisocyanate (HDI), and an isocyanate group-capped PCL diol (PCL-NCO) was synthesized. The chemical structure of the PCL-NCO was characterized by Fourier transform infrared spectroscopy to determine the presence of the isocyanate groups. A PLA/PCL blend with a PCL content of 50 wt% was used as a polymer matrix, and the as-prepared PCL-NCO was added into the polymer matrix via melt blending. The influence of the PCL-NCO modifier on thermo-responsive cyclic shape memory performance of the 3D printed PLA/PCL blend was studied. On this basis, PLA/PCL nanocomposites were prepared by adding the CNC-OMMT hybrid nanofillers with different contents, and the morphology, thermal, dynamic mechanical, cyclic shape memory, and mechanical properties of the 3D printed PLA/PCL nanocomposites were investigated. In addition, thermo-responsive shape memory behavior of the 3D printed SMP product based on the nanocomposite was verified under realistic conditions. This work aimed to improve the thermo-responsive cyclic shape memory performance of the 3D printed PLA/PCL blend using the PCL-NCO as a compatibilizer for the polyester blend system; furthermore, the CNC-OMMT hybrid nanoparticles were used as reinforcing nanofillers to enhance crystallization and mechanical properties of the 3D printed nanocomposites. This work provides a novel and effective approach for the fabrication of high-performance functional polymer nanocomposites, which have great application potential in advanced fields such as sensors and actuators.

## 2. Materials and Methods

Polycaprolactone diol (PCL diol) with an average molecular weight of 2000 g/mol and a hydroxyl value of 55.67 mg potassium hydroxide (KOH)/g was purchased from the Hunan Juren Chemical New Material Technology Co., Ltd. (Yueyang, China). Hexamethylene diisocyanate (HDI), toluene, and methanol were all purchased from Shanghai Macklin Biochemical Co., Ltd. (Shanghai, China). Bleached softwood kraft pulp, containing cellulose (92.1 wt%), hemicellulose (3.6 wt%), lignin (0.2 wt%), and a small amount of ash and moisture, was purchased from Shandong Daoxin New Materials Co., Ltd. (Weifang, China). Sodium-montmorillonite (MMT) with a cation exchange capacity of 90 mmol/100 g was purchased from Zhejiang Fenghong Clay Chemical Co., Ltd. (Huzhou, China). Sulfuric acid (98 wt%) and cetyltrimethylammonium bromide were purchased from Tianjin Damao Chemical Reagent Factory (Tianjin, China). PLA pellets (Ingeo^TM^ 4032D) with a melt flow index (MFI) of 7 g/10 min (210 °C, 2.16 kg) were purchased from Nature Works LLC (Minneapolis, MI, USA). PCL pellets (Capa^TM^ 6500) with an MFI of 5.9–7.9 g/10 min (160 °C, 2.16 kg) were purchased from Perstorp UK Ltd. (Malmö, Sweden).

### 2.1. Modification of PCL Diol

The PCL diol was modified with HDI to realize isocyanate group end capping of PCL diol molecular chains. Briefly, 20 g of PCL diol (hydroxyl group content was about 0.02 mol according to the hydroxyl value) was first put into a three-necked flask, and then 200 mL toluene was added and stirred slowly at 80 °C to dissolve the PCL diol while refluxing with a condenser tube. Based on the molar ratio (1:2) of the hydroxyl group to the isocyanate group, 0.02 mol HDI, containing 0.04 mol isocyanate groups, was dispersed in 20 mL toluene. The formed solution was added dropwise into the above PCL diol solution using a syringe, and the mixture was kept stirring at 80 °C for 6 h. After the reaction was completed and the solution was cooled, the product was precipitated with the addition of methanol, and then dissolved in toluene. This process was repeated three times to remove the unreacted HDI. The resultant solution was cast in a glass petri dish and left for 12 h at room temperature. After most of the solvent was volatilized, the product was placed in a vacuum drying oven and dried at 40 °C for 24 h to eliminate excess solvent. Finally, the isocyanate group-capped PCL diol was obtained and labeled as PCL-NCO.

### 2.2. Synthesis of CNC-OMMT Hybrid Nanofillers 

The synthesis procedure of the CNC-OMMT hybrid nanofillers followed the steps used in our previous work [27]. In brief, the CNC was extracted from the softwood kraft pulp by sulfuric acid hydrolysis, and the OMMT was prepared via organic modification of the MMT with CTAB. Then, aqueous CNC suspension was mixed with aqueous OMMT suspension, wherein the mass ratio of CNC to OMMT was 1:1. In this process, the negatively charged CNC and the positive charged OMMT were electrostatically self-assembled in aqueous solution, and the CNC-OMMT hybrid nanofillers were prepared after freeze-drying.

### 2.3. Preparation of 3D Printing Filaments 

The 3D printing filaments used for FFF were prepared by a twin screw extruder with a screw diameter of 16 mm and a screw length-diameter ratio of 40:1 (Polylab OS, Haake, Karlsruhe, Germany). Before extrusion, PLA and PCL pellets were premixed with a mass ratio of 50:50. Then, the as-prepared PCL-NCO modifier was blended with the mixed pellets. The processing temperature range was set at 160–190 °C. In the process of wire drawing, the diameter of the filaments was controlled at 1.75 ± 0.05 mm with a screw speed of 50 rpm. The resulting PLA/PCL/PCL-NCO blends were referred to as PNx (x = 2%, 4%, 6%, and 8%), where x represented the mass percentage of the PCL-NCO in the blends. For example, the mass ratio of PLA, PCL, and PCL-NCO in PN2 was 50:50:2. The blend without the PCL-NCO was referred to as PN0. Furthermore, based on the PN4 system, the CNC-OMMT hybrid nanofillers were incorporated into the PN4 matrix, and resulting nanocomposites were referred to as PN4-y (y = 0.25%, 0.5%, 0.75%, and 1%), where y represented the hybrid nanofiller content.

### 2.4. Fabrication of 3D Printed Specimens

The 3D printed specimens were fabricated using a FFF printer (S1 Architect 3D, Guoguang Instruments, Guangzhou, China). The specific operations were as follows: 3D models of the specimens were first designed by drawing software and then exported to files with STL format. The STL files were converted to G-code by slicing software, and the G-code was imported into the printer with an SD card. A series of printing parameters (including nozzle temperature, printing speed, layer thickness, etc.) were set as shown in Table 1. After the 3D printing filaments were fed into the hot nozzle of the printer, the 3D printed specimens were manufactured according to the path parsed by the G-code.

### 2.5. Characterization Methods

Fourier transform infrared (FTIR) spectroscopy: FTIR spectra of film samples were measured with a Fourier transform infrared spectrometer (VERTEX 70, Bruker, Karlsruhe, Germany). The scanning was carried out in total reflection mode with a wavenumber range of 4000–400 cm^−1^ and a spectral resolution of 4 cm^−1^.

Differential scanning calorimetry (DSC): DSC scanning was performed on a differential scanning calorimetry (204 F1, NETZSCH, Selb, Germany) with a heating rate of 10 °C/min. The samples of 5–10 mg were tested under nitrogen atmosphere at a gas flow of 30 mL/min. The crystallization and melting data were obtained from the cooling scan and the second heating scan, respectively. Crystallinity (*Xc*) of the sample was calculated using Equation (1) [29],

(1) $$X_{c}=\frac{\Delta H_{m}-\Delta H_{cc}}{\Delta H_{0}\times w_{p}}\times 100\%$$

where Δ*H_m_* and Δ*H_cc_* are the melting enthalpy and cold crystallization enthalpy, respectively, and Δ*H*_0_ is the melting enthalpy of 100% crystalline polymer (93.7 J/g for PLA and 136 J/g for PCL) [30]. In particular, the value of Δ*H_cc_* for PCL is equal to 0. The symbol *w_p_* is weight percentage of the corresponding polymer component.

Scanning electron microscope (SEM): Morphology of the nanocomposites was observed with a field-emission SEM (Nano 430, FEI, Eindhoven, Holland). Before observation, the cross-section of the sample was sprayed with gold.

Dynamic mechanical analysis (DMA): DMA was performed using a dynamic mechanical analyzer (Q800, TA Instruments, New Castle, DE, USA) with a heating rate of 2 °C/min in a temperature range of −70–120 ℃ at a frequency of 1 Hz and a strain of 0.05%.

**Figure 1:**
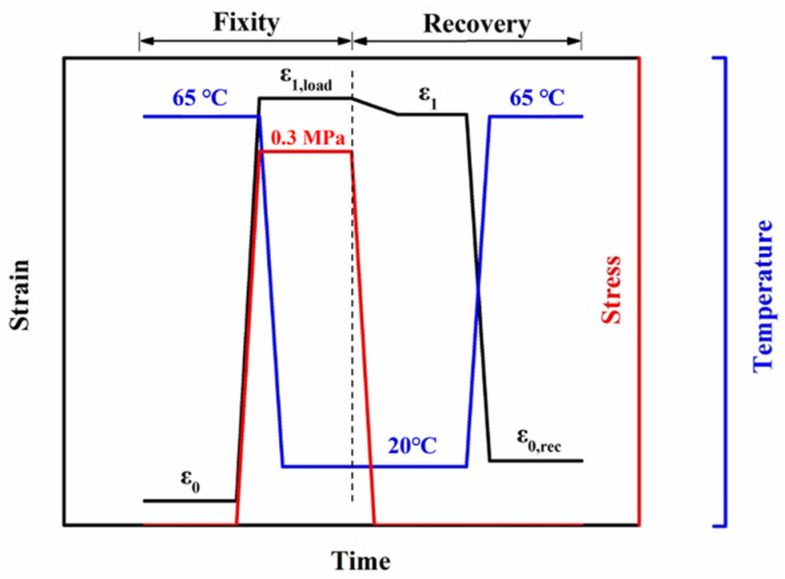
The procedure of the thermo-responsive shape memory test.

Thermo-responsive shape memory properties: A thermo-responsive shape memory test was conducted on the dynamic mechanical analyzer in stress-controlled tensile mode. The specific procedure of thermo-mechanical programming referred to our previously published report [16], as shown in Figure 1.

First, a 3D printed specimen with a dimension of 40 mm (length) × 4 mm (width) × 1 mm (thickness) was heated to 65 °C and held for 3 min, and the initial strain was denoted as *ε*_0_; then, the specimen was subjected to a uniaxial tensile stress of 0.3 MPa. With the generation of a temporary strain, the specimen was rapidly cooled to 20 °C and held at this temperature and tensile stress for 3 min to fix the temporary shape, and the strain was denoted as *ε*_1,*load*_. After removing the stress, the specimen was kept at 20 °C for 3 min, and the temporary strain was denoted as *ε*_1_. Finally, the specimen was reheated to 65 °C for strain recovery and held for 20 min, and the final strain was denoted as *ε*_0,*rec*_. The above thermo-mechanical programming was repeated three times while recording the corresponding strain data. It should be noted that the initial strain in the next cycle was the recovery strain in the previous cycle. Shape fixation rate (*R_fn_*) and shape recovery rate (*R_rn_*) were calculated by Equations (2) and (3), respectively. The symbol n represents the number of cycles.

(2) $$R_{fn}=\frac{\varepsilon_{n}-\varepsilon_{n-1}}{\varepsilon_{n,load}-\varepsilon_{n-1}}\times 100\%$$

(3) $$R_{rn}=\frac{\varepsilon_{n,load}-\varepsilon_{n-1,rec}}{\varepsilon_{n,load}-\varepsilon_{n-1}}\times 100\%$$

Mechanical properties: A uniaxial tensile test (type 1BA, ISO-527) and three-point bending test (ISO-178) were conducted on a universal electronic testing machine (Z010, Zwick/Roell, Ulm, Germany) with a testing rate of 5 mm/min. An impact test (ISO-180) of the 3D printed specimen (V-notch) was performed using a cantilever beam impact testing machine (5113, Zwick/Roell, Germany) with a 2.75 J pendulum. Each group of specimens was measured at least five times, and the reported value was the average of the measurement results.

## 3. Results and Discussion

### 3.1. Characterization of PCL-NCO and Reactive Mechanism

**Figure 2:**
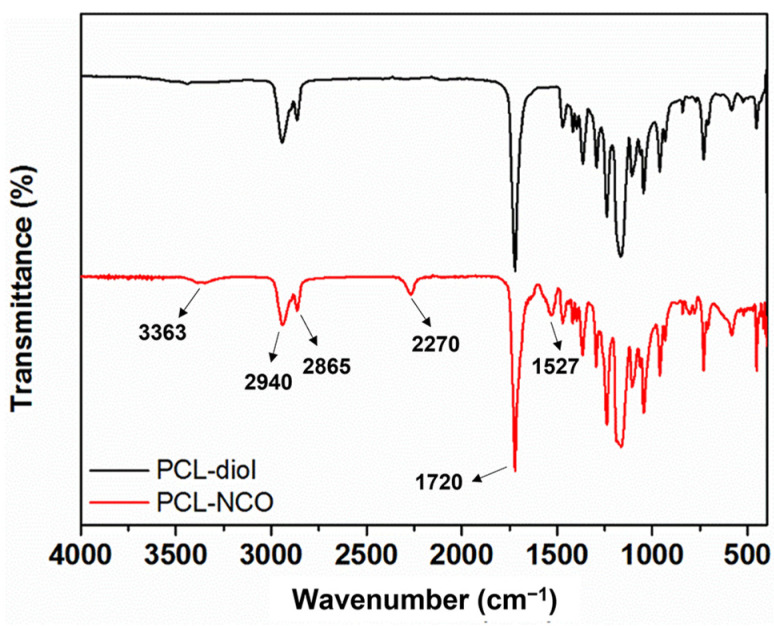
FTIR spectra of the PCL diol and PCL-NCO.

An FTIR spectroscope was used to characterize the chemical structure of PCL-NCO. The FTIR spectra of the PCL diol and PCL-NCO are shown in Figure 2. An absorption peak at 1720 cm^−1^ in the spectra of the two samples corresponded to stretching vibration of carbonyl group, and two absorption peaks at 2940 cm^−1^ and 2965 cm^−1^ were attributed to asymmetric and symmetric stretching vibrations of ₋CH_2_- chains, respectively. This indicated that the main chain structure of the PCL-NCO did not change after HDI modification. However, the difference between the spectra of the PCL diol and PCL-NCO can be clearly distinguished. For example, a small absorption band existing around 3363 cm^−1^ only in the spectrum of the PCL-NCO was assigned to -N-H stretching vibration of newly formed urethane bonding; a characteristic peak at 1527 cm^−1^ presented in the PCL-NCO sample was associated with -C-N- stretching vibration and out-of-plane bending vibration of the urethane bonding [31]. The appearance of these new peaks confirmed that isocyanate groups of the HDI reacted with hydroxyl groups at the end of PCL diol chains to form urethane bonding. It is noteworthy that the PCL-NCO sample also had a new peak at 2270 cm^−1^, corresponding to the stretching vibration of the isocyanate groups [32], which indicated that there existed unreacted isocyanate groups at the end of the PCL-NCO chains. The FTIR analysis showed that the PCL diol was successfully modified by the HDI and capped with the isocyanate groups.

**Figure 3:**
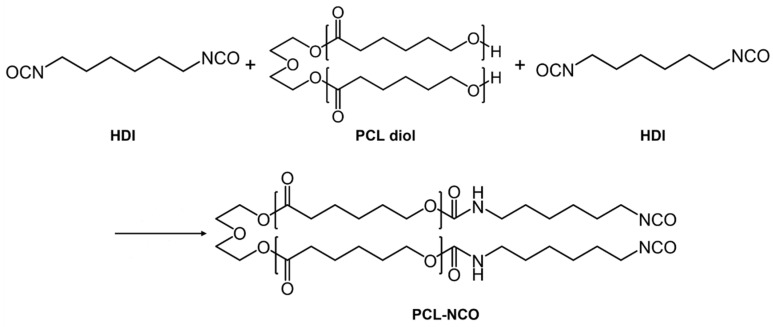
Schematic diagram of the reaction mechanism of the HDI-modified PCL diol.

Based on the above results, the reaction mechanism of the HDI-modified PCL diol was proposed, as shown in Figure 3. In order to achieve the capping of isocyanate groups, the molar ratio of the HDI to PCL diol was controlled as 2:1, that is, the molar ratio of isocyanate group content in the HDI to hydroxyl group content in the PCL diol was 2:1. Due to high reactivity of the isocyanate groups with hydroxyl active sites on the short chains of the PCL diol, the PCL diol can react with the HDI to form urethane bonding.

In addition to the FTIR spectra, thermal properties of the PCL-NCO were also studied using DSC and thermal gravimetry (TG) analysis (Figures S1 and S2, Supporting Information), and corresponding data are summarized in Tables S1 and S2, respectively. It was found that the PCL diol showed a distinct melting crystallization peak in the cooling scan, indicating that the PCL diol had strong crystallization ability. However, the PCL diol had a melting point of only 45.9 °C due to its low molecular weight. From the melting enthalpy data derived from the second heating scan, it can be calculated that the crystallinity of the PCL diol was 58.7%. After the HDI modification, the melting point and crystallinity of the PCL-NCO were reduced to 44.4 °C and 37.4%, respectively. This is probably due to the weakened molecular chain regularity and chain mobility of the PCL-NCO. In terms of the thermal stability, the onset thermal decomposition temperature and maximum thermal decomposition temperature of the PCL-NCO were 334.9 °C and 420.2 °C, respectively, which were not much different from those of the PCL diol. This also indicated that the PCL-NCO had good thermal stability at around 190 °C (the processing temperature used in this work) without thermal degradation.

### 3.2. Cyclic Shape Memory Properties of PLA/PCL Blends Modified by the PCL-NCO

**Figure 4:**
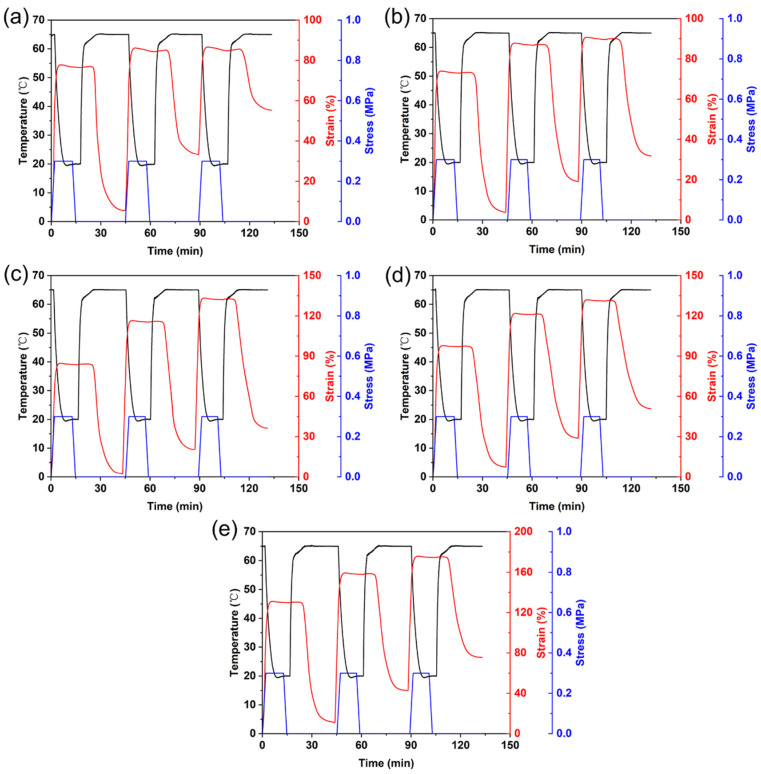
Temperature, strain, and stress curves of the PCL-NCO modified PLA/PCL blends during the three shape memory cycles: (**a**) PN0, (**b**) PN2, (**c**) PN4, (**d**) PN6, and (**e**) PN8.

Thermo-responsive cyclic shape memory behaviors of 3D printed PLA/PCL blends with different PCL-NCO contents are shown in Figure 4, and the calculated values of R_fn_ and R_rn_ are listed in Table 2. The results showed that the R_fn_ values of the PN0 sample were all above 95% in the three thermo-mechanical cycles, suggesting that the blend had good shape fixation performance. This is attributed to the fact that reversible phase PCL with fine crystallization ability enabled the temporary shape to be fixed in time by rapid crystallization during the cooling process. However, the R_r2_ and R_r3_ of the PN0 were only 65.48% and 58.72%, respectively, although its R_r1_ reached 92.66%. In other words, the cyclic shape memory performance of the PN0 decreased dramatically as the number of thermo-mechanical cycles increased. It has been known that in the PLA/PCL blend system, the physical crosslinking formed via chain entanglements between PLA and PCL chains at the phase interface played a crucial role in shape recovery [16]. However, the interfacial entangled chains were likely disentangled during the cycles, leading to an increased degree of irreversible slippage of the PCL chains under the programming stress. As a result, the cyclic shape recovery performance of the PN0 was degraded.

It is worth noting that the cyclic shape memory effect of the PLA/PCL blend was markedly enhanced with the addition of the PCL-NCO modifier. For instance, the R_r1_, R_r2_ and R_r3_ of the PN4 reached 97.14%, 85.97%, and 84.30%, respectively, which were increased by 4.8%, 31.3%, and 43.6% in comparison with the PN0. In addition, it can be seen that the R_rn_ of the 3D printed specimens decreased gradually with increasing PCL-NCO content when the PCL-NCO content was greater than 4%. Since the PCL-NCO and the PCL belong to the same substance, the PCL-NCO can be regarded as part of the PCL phase. With the increase of PCL phase content, the ability of the fixed phase PLA to constrain the viscous flow of the PCL chains was reduced. Therefore, the cyclic shape recovery performance of the blends with higher PCL-NCO contents became worse.

In order to elucidate the role of the PCL-NCO in the PLA/PCL blend, the modified blends were characterized using FTIR spectroscopy (Figure S3). It was found that the FTIR spectrum of the PN0 was a simple superposition of those of neat PLA and neat PCL, showing the characteristic absorption peaks for neat PLA and neat PCL. For the PCL-NCO modified blends, the stretching vibration peak of the PCL-NCO at 2270 cm^−1^ disappeared, indicating that the isocyanate groups reacted with the polymer matrix. Moreover, the spectra of the modified blends showed a very weak peak of -C-N- in the urethane bonding at 1527 cm^−1^ with increasing PCL-NCO content. Accordingly, the action mechanism of the PCL-NCO in the polyester blends was proposed, as shown in Figure S4. It was reasonable to consider the PCL-NCO as a compatibilizer for the PLA/PCL blend. During the melt extrusion process, the two isocyanate groups at the end of the PCL-NCO chains were likely to react randomly with the hydroxyl groups at the end of the PLA and PCL chains, respectively, to generate carbamate groups [23]. The resulting covalent sites enhanced the interface and reduced the irreversible slippage of the PCL chains in the thermo-mechanical cycles, thus effectively improving the cyclic shape memory performance of the blends.

### 3.3. Morphologies of PLA/PCL Nanocomposites

**Figure 5:**
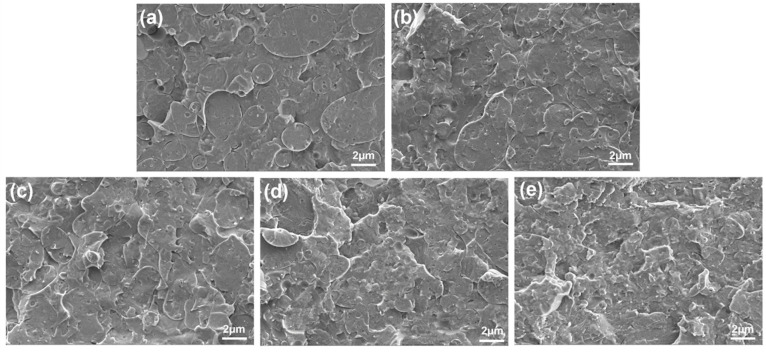
SEM images of the PLA/PCL nanocomposites: (**a**) PN4, (**b**) PN4-0.25, (**c**) PN4-0.5, (**d**) PN4-0.75, and (**e**) PN4-1.

In view of better cyclic shape memory properties, the PN4 blend was used as a polymer matrix and the CNC-OMMT hybrid nanofillers were incorporated into the PN4 to prepare nanocomposites. The morphologies of the PLA/PCL nanocomposites were observed via SEM, as shown in Figure 5. In our previous study, it was found that the PCL phase was distributed in the PLA matrix as a semi-continuous structure in the PLA/PCL blend containing 50 wt% PCL (i.e., the PN0 in this work) [16]. However, phase structure of the PN4 was significantly transformed with the introduction of the PCL-NCO. It can be seen that the PCL phase was dispersed as spherical droplets with a diameter size of approximately 1–5 μm. This may be due to the aforementioned compatible effect of the PCL-NCO, which led to the tendency of the PCL phase to coalesce together and decrease in free energy of the phase interface [33]. This also explained the improvement of the cyclic shape memory performance of the PLA/PCL blend modified by the PCL-NCO from the perspective of its microstructure. The PCL phase was entirely surrounded by the PLA phase, so the PCL chains only moved in a limited area. Under this condition, the interfacial interaction effectively restricted the viscous flow of the PCL chains, and therefore the degree of irreversible slippage was reduced in the shape memory testing cycles.

When a small amount of the hybrid nanofillers (such as 0.25% or 0.5%) was added, phase structure of the nanocomposites did not change much, but the phase interface gradually became distorted. Meanwhile, the original spherical droplet PCL phase appeared to be squeezed to become irregular. As the hybrid nanofiller content continued to increase, the phase interface became blurred, and the size of the PCL phase decreased. As reported in the literature, nanoparticles can stabilize phase morphology of polymer blends via reducing interfacial tension [34]. Additionally, the overall phase structure of the nanocomposites became rougher with increasing hybrid nanofiller content, but no evident nanofiller agglomeration was observed. This indicated that on the one hand, the hybrid nanofillers were likely to be distributed in both PLA and PCL phases; on the other hand, the hybrid nanofillers had adequate dispersion in the polymer matrix.

### 3.4. Thermal Properties of PLA/PCL Nanocomposites

**Figure 6:**
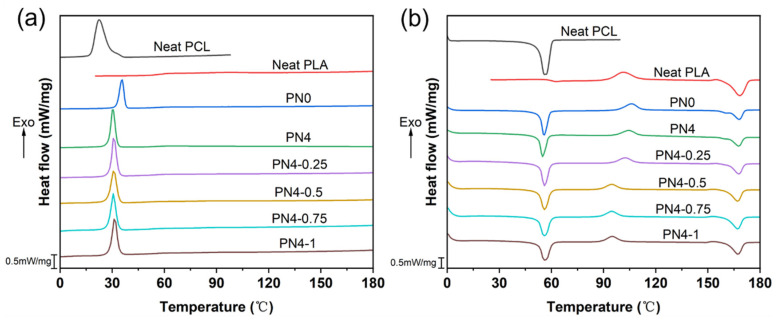
DSC curves measured for the PLA/PCL nanocomposites: (**a**) the cooling scans, (**b**) the second heating scans.

Figure 6 shows DSC curves measured for the PLA/PCL blend and PLA/PCL nanocomposites, and the corresponding thermal parameters of PCL and PLA components are summarized in Table 3 and Table 4, respectively. Melting crystallization temperature (T_mc_) and melting crystallization enthalpy (∆H_mc_) were derived from the cooling scan, while cold crystallization temperature (T_cc_), cold crystallization enthalpy (∆H_cc_), melting temperature (T_m_), and melting enthalpy (∆H_m_) were obtained from the second heating scan.

Neat PCL exhibited adequate crystallization performance with a crystallinity of 44.9%, whereas the crystallinity of neat PLA was only 5.2% due to the slow crystallinity rate. The PCL component always maintained good melting crystallization ability in the blends (PN0, PN4) and nanocomposites, which was the reason for the excellent shape fixation properties of the PLA/PCL blend system. Specifically, the PCL component, as the reversible phase, crystallized rapidly from the molten state during the cooling process, so that the temporary deformation could be fixed in time. After adding the PCL-NCO modifier, the T_mc, PCL_ and X_c, PCL_ of the PN4 decreased slightly. This may be because the interfacial compatibilization effect of the PCL-NCO made some of the PCL chains miscible with the PLA phase at the interface. Furthermore, the X_c, PCL_ increased gradually from 39.9% of the PN4 to 47.0% of the PN4-1, indicating that the incorporation of the CNC-OMMT hybrid nanofillers was beneficial to the crystallization of the PCL phase. As for the PLA component, due to the slow crystallization rate, the PLA phase showed apparent cold crystallization behavior around 100 °C during the second heating process. However, compared to the PN0, the T_cc, PLA_ of the PN4 decreased and its X_c, PLA_ increased, which was attributed to the nucleation effect of the PCL component miscible at the interface on the PLA crystallization [35]. Notably, with the addition of the hybrid nanofillers, the T_cc, PLA_ of the nanocomposites was significantly reduced, while the X_c, PLA_ increased from 4.3% of the PN4 to 23.1% of the PN4-1. These results indicated that the hybrid nanofillers greatly enhanced crystallization of the nanocomposites. The main reason for this was that the well-dispersed hybrid nanofillers were efficient heterogeneous-nucleating agents and improved the crystallization rate [28].

It was also found that the PCL-NCO and hybrid nanofillers had no significant impacts on the T_m, PCL_ and T_m, PLA_. With regard to the polymer blend system, the PLA component with a high melting point generally played a role in maintaining the permanent shape in the shape memory behavior, while the PCL component with a low melting point changed the temporary shape through flow-freezing transition of molecular chains. In other words, the internal stress stored in the frozen molecular chains was the driving force for the shape recovery, which can be released by re-flowing of the chains [36]. The wide melting temperature window between the PCL and PLA phases provided an essential prerequisite for the thermo-responsive SME.

### 3.5. Dynamic Mechanical Properties of PLA/PCL Nanocomposites

**Figure 7:**
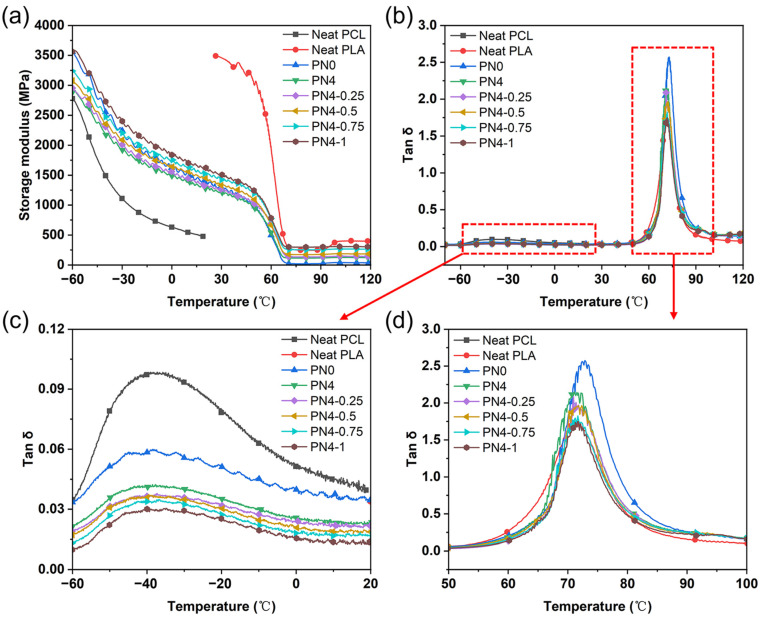
DMA curves measured for the PLA/PCL nanocomposites: (**a**) storage modulus, tan δ in the temperature range of (**b**) −80–120℃, (**c**) −60–20℃, and (**d**) 50–100℃.

DMA curves of the PLA/PCL blend and corresponding nanocomposites are shown in Figure 7. DMA data such as storage modulus E’, Tan δ peak temperature, and Tan δ peak value are summarized in Table 5. It can be seen from Figure 7a that neat PCL was in a rubbery state at room temperature, and its E’ was only 478 MPa, while that of neat PLA was up to 3489 Mpa. Moreover, the E’ of the PN0 was 1322 Mpa, which was between the storage modulus of the PLA and PCL components. The E’ of the PN4 decreased further with the addition of the PCL-NCO. However, the E’ of the PLA/PCL nanocomposites increased with increasing CNC-OMMT content. For example, the E’ of the PN4-1 was 1550 Mpa, which was 25.6% higher than that of the PN4. The results indicated that the hybrid nanofillers had enhancement effects on the PLA/PCL blend.

The Tan δ curves in Figure 7b show that the PLA/PCL blend system had two distinct independent phase transition regions, indicating that the blend was a phase separation structure, which also provided a prerequisite for the thermally responsive SME. According to the Tan δ curves, it can be concluded that the PLA/PCL blend system underwent two phase transition stages in the temperature range of −60–120 °C. The first stage was the glass transition of the PCL phase at −60–20 °C, and corresponding glass transition temperature (T_g_) was about −39.2 °C. The second stage was mainly manifested in the glass transition of the PLA phase at 50–100 °C, and corresponding T_g_ fluctuated in the range of 70–73 °C. It should be noted that the PCL phase actually experienced a transition from a rubbery state to a viscous state at this stage, which may be covered by the glass transition of the PLA phase. These two stages led to a sharp decline in the E’ of the blend system. However, the PLA chains remained in a glassy/rubbery state during the shape memory cycles, acting as a permanent network in the SME.

It is well known that the loss factor Tan δ can be considered an indicator of polymer chain mobility [37]. As shown in Figure 7c, the Tan δ peak value and peak area of the PN0 decreased significantly at −60–20 °C compared to neat PCL. This was due to the fact that the glassy PLA chains restricted the movement of PCL chain segments. Moreover, when the PCL-NCO was added, the covalent sites formed at the phase interface weakened the mobility of the PCL chain segments, resulting in a further decrease in the Tan δ peak value of the PN4. It was also found that the Tan δ peak value of the PLA/PCL nanocomposites decreased with the increase of CNC-OMMT content, which may be because the uniformly dispersed rigid nanoparticles hindered the movement of the PCL chain segments. Similar rules can be detected from the Tan δ curves appearing in the glass transition region of the PLA phase. In the temperature range of 50–100 °C (Figure 7d), the Tan δ peak value and peak area of the PN0 were apparently increased compared with those of neat PLA due to the intense viscous flow of the PCL chains. Furthermore, the T_g, PLA_ increased from 71.5 °C of neat PLA to 72.9 °C of the PN0. This was attributed to the large number of viscous flow PCL chains that blocked the movement of PLA chain segments, leading to a rise of demand temperature for the PLA phase transition. Notably, compared with the PN0, the Tan δ peak value and peak area of the PN4 decreased instead, and the T_g, PLA_ of the PN4 was shifted to the left of 70.6 °C, which further confirmed the interfacial compatibilization effect of the PCL-NCO on the PLA/PCL blend system. With regard to the PLA/PCL nanocomposites, the well-dispersed nanofillers weakened chain mobility, so the Tan δ peak value showed a downward trend.

In conclusion, the PCL-NCO modifier can effectively improve the phase interface of the PLA/PCL blend system, and the CNC-OMMT hybrid nanofillers had reinforcement effects on the blend, but the hybrid nanofillers inhibited the chain mobility.

### 3.6. Cyclic Shape Memory Properties of PLA/PCL Nanocomposites

**Figure 8:**
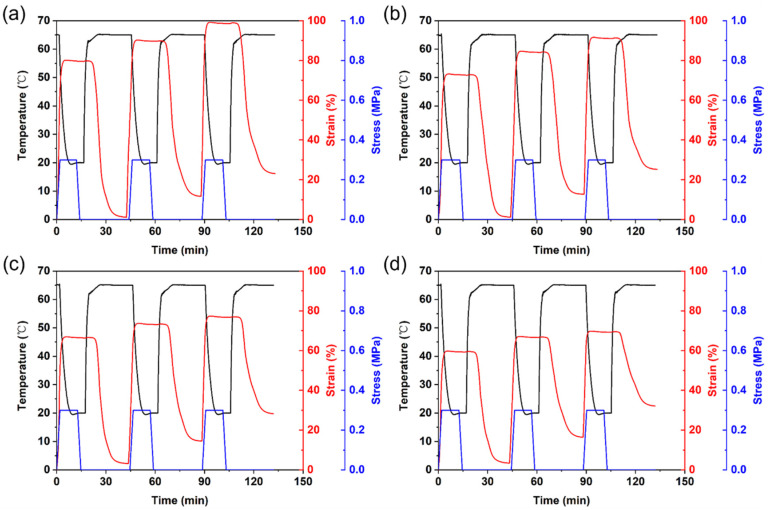
Temperature, strain, and stress curves of the PLA/PCL nanocomposites during the three shape memory cycles: (**a**) PN4-0.25, (**b**) PN4-0.5, (**c**) PN4-0.75, and (**d**) PN4-1.

Thermo-responsive cyclic shape memory behaviors of the 3D printed PLA/PCL nanocomposites with different CNC-OMMT contents are shown in Figure 8, and the calculated values of R_fn_ and R_rn_ are listed in Table 6. Similar to the PLA/PCL blend, the PLA/PCL nanocomposites also showed excellent shape fixation properties during the three thermo-mechanical cycles, because the reversible phase PCL still maintained adequate crystallization ability in the nanocomposites. Regarding the shape recovery, the R_r1_, R_r2_, and R_r3_ of the PN4-0.25 reached 98.50%, 88.07%, and 86.81%, respectively, which was improved compared to the PN4. The PN4-0.5 also had a slight improvement in the shape recovery performance. A similar study has found that dispersed hydroxyapatite (HA) particles can act as an additional fixed phase to reduce chain mobility in shape memory behavior of PLA [38]. Like the HA, a small amount of uniformly dispersed hybrid nanofillers also acted as the fixed phase, thus limiting irreversible slippage of the PCL chains to a certain extent. However, the shape recovery performance of the nanocomposites became worse with increasing hybrid nanofiller content. For instance, the R_r1_, R_r2_, and R_r3_ of the PN4-1 were reduced to 94.02%, 80.42%, and 70.66%, respectively. The results indicated that too many hybrid nanoparticles (>0.5%) had adverse effects on the shape recovery property. The DMA analysis revealed that the well-dispersed CNC-OMMT restricted chain mobility. Yet, the driving force for the shape recovery was to release the internal stress stored in the temporary network through the movement of the PCL chains. That is to say, a large number of the rigid hybrid nanoparticles distributed in the polymer matrix were likely to hinder entropy elastic recovery of the PCL chains [39], therefore weakening the shape recovery property. In general, the cyclic shape memory performance of the nanocomposites can be enhanced only with an appropriate amount of the CNC-OMMT nanofillers.

**Figure 9:**
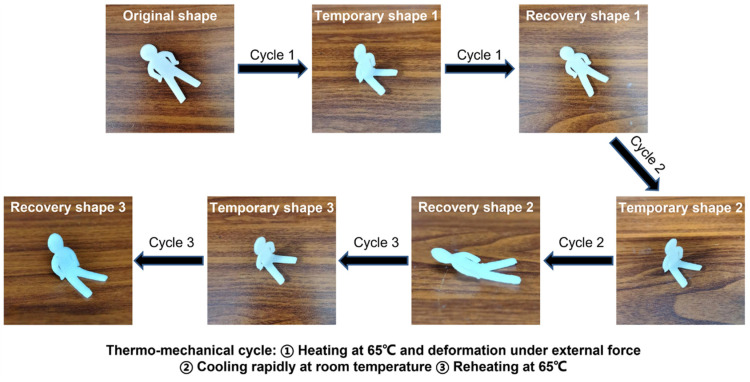
Thermo-responsive cyclic shape memory behavior of a 3D printed doll product.

In order to further evaluate the cyclic shape memory performance of the 3D printed PLA/PCL nanocomposites, a PN4-1 sample-based doll product was fabricated by the FFF. Figure 9 shows thermo-responsive cyclic shape memory behavior of the 3D printed doll under practical conditions. First, the doll was heated to 65 °C and kept at that temperature for 5 min; then the “lying” doll was transformed into a temporary shape of a “sitting” doll via external force. After rapid cooling to room temperature, the temporary shape of the doll was fixed without external force. Later, the doll with the temporary shape was reheated to 65 °C, where it was observed that the doll gradually stretched to the “lying” state and eventually recovered to its original shape. After three cycles of the thermo-mechanical manipulation, the final shape of the doll became a “semi-lying” position. It was observed that the shape of the doll was unable to be completely restored as the number of cycles increased. The thermo-responsive cyclic shape memory behavior of the 3D printed doll product under practical conditions was basically consistent with the DMA result.

### 3.7. Mechanical Properties of PLA/PCL Nanocomposites

**Figure 10:**
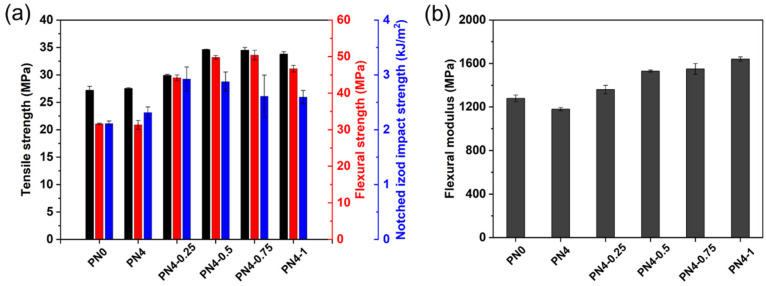
Mechanical properties of the PLA/PCL nanocomposites: (**a**) tensile strength, flexural strength, and notched impact strength, and (**b**) flexural modulus.

Figure 10 shows the mechanical properties of the 3D printed PLA/PCL nanocomposites. Tensile strength, flexural strength, notched impact strength, and flexural modulus of the PN0 were 27.2 MPa, 31.6 MPa, 2.11 kJ/m^2^, and 1280 MPa, respectively. As can be seen from the trend of the mechanical properties, compared with the PN0, tensile and flexural strength of the PN4 had no significant changes, but the notched impact strength underwent a slight increase; however, the flexural modulus of the PN4 decreased, which was related to the addition of the PCL-NCO with low molecular weight. It was also found that the CNC-OMMT hybrid nanofillers effectively enhanced the mechanical properties of the nanocomposites. For instance, tensile, flexural, and notched impact strength of the PN4-0.5 were 34.6 MPa, 49.8 MPa, and 2.87 kJ/m^2^, respectively, which were increased by 25.8%, 37.1%, and 24.2% compared to the PN4. Furthermore, the flexural modulus of the nanocomposites also increased with the increase of nanofiller content. It was believed that the reinforcement of the mechanical properties was attributed to the uniform distribution of the hybrid nanofillers in the polymer matrix [28]. The results showed that the hybrid nanoparticles had obvious reinforcement effects on the PLA/PCL nanocomposites even if the nanofiller content was less than 1%. In summary, when the PCL-NCO content was 4% and the CNC-OMMT content was 0.5%, the modified PLA/PCL nanocomposites not only maintained favorable cyclic shape memory performance (R_f_ > 99% and R_r_ > 85%), but also had fine mechanical performance.

## 4. Conclusions

The stress-controlled DMA in tensile mode indicated that the SME of the PN0 blend, which mainly depended on the physical interfacial interaction, weakened dramatically as number of the thermo-mechanical cycles increased. The PCL-NCO modifier was prepared by introducing isocyanate groups at the end of PCL diol chains with the aid of HDI. The FTIR analysis confirmed the formation of urethane bonding in the PCL-NCO, together with the presence of unreacted isocyanate groups. As a compatibilizer for the PLA/PCL blend system, the PCL-NCO reacted with the PLA and PCL to form covalent sites during the melt blending process, thus enhancing the interface of the blend, as demonstrated by the DMA results. The cyclic shape recovery performance of the modified PLA/PCL blends was significantly improved.

Further, the CNC-OMMT hybrid nanofillers were incorporated into the modified PN4 blend and the PLA/PCL nanocomposites were prepared by FFF 3D printing. SEM observations showed that the hybrid nanofillers dispersed well in the polymer matrix and made the phase structure of the nanocomposites become rough. Accordingly, the mechanical properties of the nanocomposites were superior to those of the blend without the hybrid nanofillers. Additionally, the crystallization performance of the nanocomposites was also enhanced due to the heterogeneous nucleation effects of the hybrid nanofillers. In terms of the shape memory performance, the R_f_ of the PN4-0.25 was above 99%, and its R_r1_, R_r2_, and R_r3_ reached 98.50%, 88.07%, and 86.81%, respectively. However, the high content of the hybrid nanofillers (>0.5%) greatly limited the chain mobility, which was not conducive to shape recovery. This was verified by observing the cyclic shape memory behavior of corresponding 3D printed product under actual conditions. In a word, the 3D printed PLA/PCL nanocomposites containing 4% PCL-NCO and 0.5% CNC-OMMT had favorable shape memory performance, crystallization, and mechanical properties. This work provided a novel and effective approach for the fabrication of high-performance functional polymer nanocomposites. It is expected that the shape memory performance and mechanical properties of the PLA/PCL nanocomposites will be further enhanced when the interfacial interaction is improved by the introduction of appropriate chemical crosslinking networks.

## Figures and Tables

**Table 1:** Three-dimensional printing parameters used in the sample preparation.

Parameter	Nozzle Diameter	Nozzle Temperature	Printing Speed	Layer Thickness	Raster Angle	Infill Density	Platform Temperature
Value	0.6 mm	190 °C	40 mm/s	0.05 mm	45°/−45°	100%	30 °C

**Table 2:** R_fn_ and R_rn_ of the PLA/PCL blends with different PCL-NCO contents.

Sample	R_f1_ (%)	R_r1_ (%)	R_f2_ (%)	R_r2_ (%)	R_f3_ (%)	R_r3_ (%)
PN0	98.25	92.66	97.72	65.48	96.55	58.72
PN2	98.65	94.89	98.76	82.31	98.49	81.79
PN4	98.87	97.14	99.08	85.97	99.01	84.30
PN6	99.23	92.59	99.26	81.15	99.14	78.72
PN8	99.04	91.50	99.09	78.67	98.84	75.35

**Table 3:** DSC data of the PCL component derived from the DSC curves.

Sample	T_mc_^a^ (°C)	∆H_mc_^b^ (J/g)	T_m_^c^ (°C)	∆H_m_^d^ (J/g)	X_c_^e^ (%)
Neat PCL	22.5	62.2	56.7	61.0	44.9
PN0	35.8	29.3	55.9	29.2	42.9
PN4	30.4	29.1	55.2	27.1	39.9
PN4-0.25	30.6	31.2	55.9	30.9	45.4
PN4-0.5	30.6	31.0	55.8	30.8	45.3
PN4-0.75	30.5	31.4	55.9	31.3	46.0
PN4-1	31.0	32.0	56.0	32.0	47.1

^a^ melting crystallization temperature; ^b^ melting crystallization enthalpy; ^c^ melting temperature; ^d^ melting enthalpy; ^e^ crystallinity.

**Table 4:** DSC data of the PLA component derived from the DSC curves.

Sample	T_cc_^a^ (°C)	T_m_ (°C)	∆H_cc_^b^ (J/g)	∆H_m_ (J/g)	X_c_ (%)
Neat PLA	101.3	168.1	30.7	35.6	5.2
PN0	106.1	168.0	16.5	17.5	2.1
PN4	104.4	167.7	13.8	15.8	4.3
PN4-0.25	101.5	167.9	13.9	17.7	8.1
PN4-0.5	93.6	167.2	12.4	21.7	19.9
PN4-0.75	93.8	167.2	12.8	23.1	22.0
PN4-1	94.0	167.4	12.8	23.6	23.1

^a^ cold crystallization temperature; ^b^ cold crystallization enthalpy.

**Table 5:** DMA data of the PLA/PCL nanocomposites derived from the DMA curves.

Sample	Storage Modulus E’ at 25 °C (MPa)	Tan δ Peak Temperature (°C)	Tan δ Peak Value
Neat PCL	478	−39.2	0.10
Neat PLA	3489	71.5	1.72
PN0	1322	72.9	2.57
PN4	1234	70.6	2.11
PN4-0.25	1283	70.9	2.08
PN4-0.5	1392	71.7	1.96
PN4-0.75	1485	71.2	1.79
PN4-1	1550	71.6	1.75

**Table 6:** R_fn_ and R_rn_ of the PLA/PCL nanocomposites with different CNC-OMMT contents.

Sample	R_f1_ (%)	R_r1_ (%)	R_f2_ (%)	R_r2_ (%)	R_f3_ (%)	R_r3_ (%)
PN4-0.25	99.15	98.50	99.24	88.07	99.24	86.81
PN4-0.5	99.17	97.98	99.19	85.80	99.26	85.23
PN4-0.75	99.26	94.75	99.17	84.05	98.98	78.14
PN4-1	99.06	94.02	99.16	80.42	98.81	70.66

## Data Availability

The data presented in this study are available on request from the corresponding author.

## Supplementary Materials

The following supporting information can be downloaded at: https://www.mdpi.com/article/10.3390/polym15061533/s1, Figure S1: DSC curves measured for the PCL diol and PCL-NCO: (a) the cooling scans, (b) the second heating scans; Ta-ble S1: DSC data derived from the measured DSC curves; Figure S2: Thermal gravimetry curves measured for the PCL diol and PCL-NCO: (a) TG curves, (b) DTG curves; Table S2: TG data derived from the measured TG and DTG curves; Figure S3: FTIR spectra of the PLA/PCL blends modified with different PCL-NCO contents; Figure S4: Schematic dia-gram of action mechanism of the PCL-NCO in the PLA/PCL blend system.
