# Supplementary material for: Fabrication of 3D Printed Polylactic Acid/Polycaprolactone Nanocomposites with Favorable Thermo-Responsive Cyclic Shape Memory Effects, and Crystallization and Mechanical Properties
Source: Polymers (Basel). 2023 Mar 20;15(6):1533. doi: 10.3390/polym15061533 (PMC10053012; doi:10.3390/polym15061533)
Supplement: Supplementary file 1 [file polymers-15-01533-s001.zip › polymers-2273422-supplementary.pdf]

Supporting Information

Fabrication of 3D printed polylactic acid/polycaprolactone nanocomposites with favorable thermo-responsive cyclic shape memory effects, and crystallization and mechanical properties

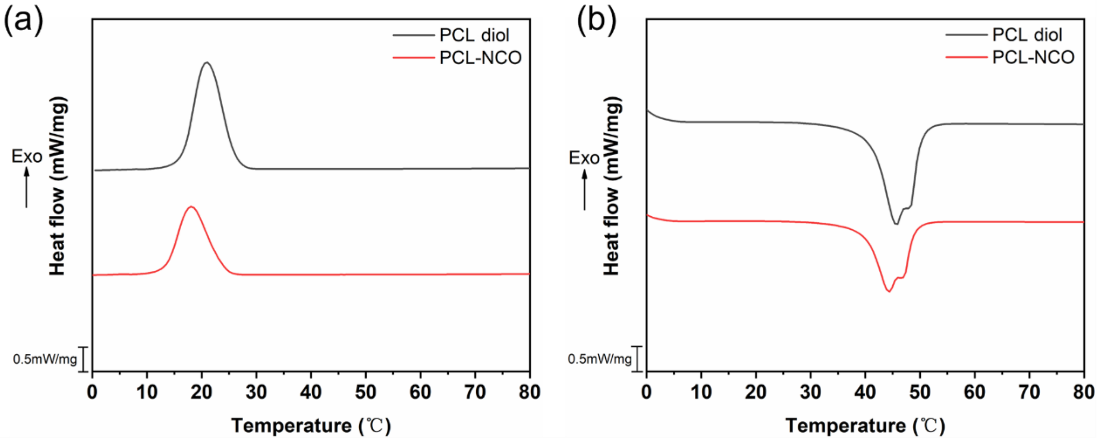

Figure S1. DSC curves measured for the PCL diol and PCL-NCO: (a) the cooling scans, (b) the second heating scans.

Table S1. DSC data derived from the measured DSC curves.

Sample	$T_{mc}{}^{a}$ (℃)	$\Delta H_{mc}{}^{b}$ (J/g)	$T_{m}{}^{c}$ (°C)	$\Delta H_{m}{}^{d}$ (J/g)	$X_{c}{}^{e}$ (%)
PCL diol	20.9	80.2	45.9	79.8	58.7
PCL-NCO	18.1	53.6	44.4	50.8	37.4

[a]melting crystallization temperature; [b]melting crystallization enthalpy; [c]melting temperature; [d]melting enthalpy; [e]crystallinity.

Thermal gravimetry (TG) analysis: The TG analysis was performed on a thermogravimetric analyzer (209 F1, NETZSCH, Germany) with a heating rate of 20℃/min under nitrogen atmosphere.

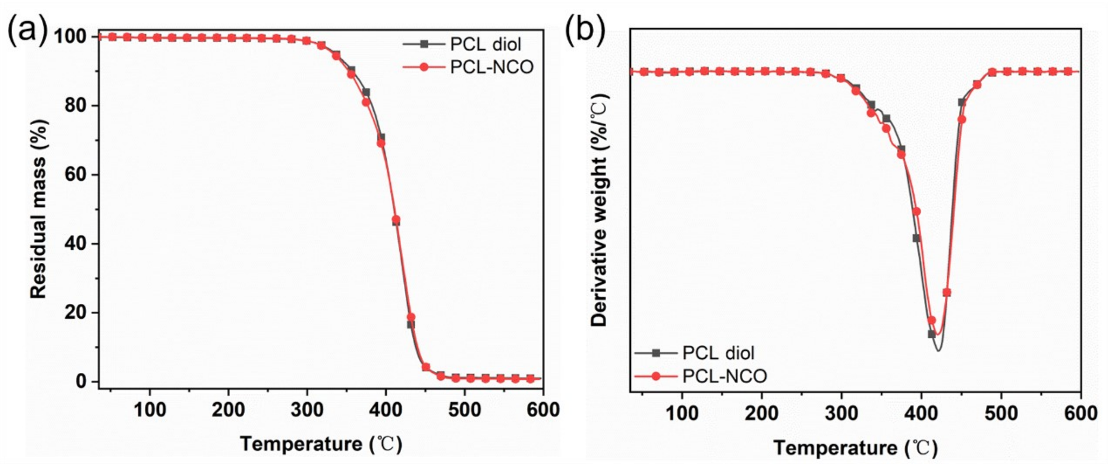

Figure S2. Thermal gravimetry curves measured for the PCL diol and PCL-NCO: (a) TG curves, (b) DTG curves.

Table S2. TG data derived from the measured TG and DTG curves.

Sample	T_{onset}[a] (℃)	T_{max}[b] (℃)	Residual mass (%)
PCL diol	336.2	421.1	0.78
PCL-NCO	334.9	420.2	1.01

[a]onset thermal decomposition temperature;

[b]maximum thermal decomposition temperature.

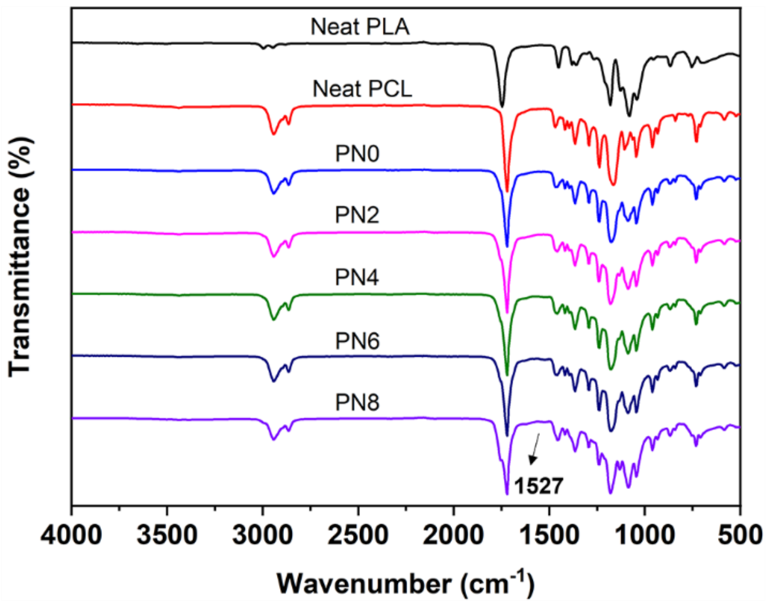

Figure S3. FTIR spectra of the PLA/PCL blends modified with different PCL-NCO contents.

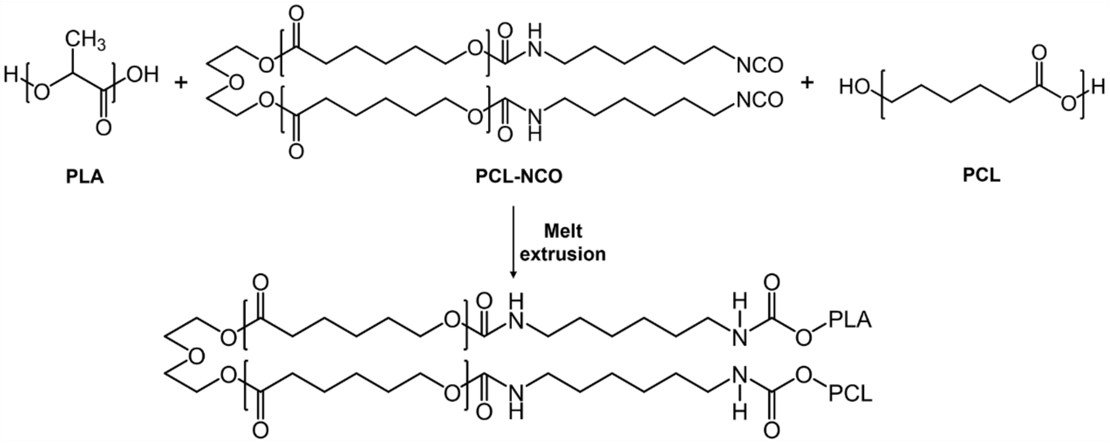

Figure S4. Schematic diagram of action mechanism of the PCL-NCO in the PLA/PCL blend system.
